# Physical Activity and Depressive Disorders in Pregnant Women—A Systematic Review

**DOI:** 10.3390/medicina55050212

**Published:** 2019-05-26

**Authors:** Daria Kołomańska, Marcin Zarawski, Agnieszka Mazur-Bialy

**Affiliations:** 1 Department of Ergonomics and Exercise Physiology, Faculty of Health Science, Jagiellonian University Medical College, Grzegorzecka 20, 31-531 Krakow, Poland; daria.kolomanska@gmail.com; 2 Department of Gynecology and Obstetrics with Gynecologic Oncology, Rydygier Hospital, Złotej Jesieni 1, 31-826 Krakow, Poland; mzarawski@gmail.com

**Keywords:** pregnancy, pregnant woman, physical activity, pregnant exercises, depression

## Abstract

*Background and Objectives:* Pregnancy is a unique period in the life of every woman. The lifestyle of a pregnant woman has a significant impact on her and her child’s health. Regular physical activity is one of the elements that help maintain normal mental and physical well-being. In pregnant women who regularly have moderate physical activity, there is a lower risk of developing obesity and overweight. Physical exercises have an impact on maintaining proper muscular tonus, reduce pain and prepare for the exertion during labour. Based on the available literature, the aim of this study was to present the impact of physical activity on depressive disorders in pregnant women. *Materials and Methods:* A review of the literature was carried out in the Medline PubMed database. The basic search terms were: “pregnancy” AND “physical activity AND depression”. The work included only English-language publications published in the years 2000–2018. *Results:* A total of 408 references were found. On the basis of an analysis of titles, abstracts and the language of publication (other than English), 354 articles were rejected, and 54 articles were fully read, of which five were rejected due to lack of access to the full version. Finally, 17 references were included in the review. *Conclusions:* Physical activity, at least once a week, significantly reduces the symptoms of depression in pregnant women and may be an important factor in the prevention of depression in this period.

## 1. Introduction

According to the WHO (World Health Organization) definition, depression is a mental health disorder that constitutes a major social problem [1]. The main symptoms of depression are sadness, loss of interest, feelings of tiredness and a loss of energy that last for a minimum of two weeks [2]. In addition, depression is accompanied by anxiety, sleep problems, changes in appetite, concentration disorders, feelings of guilt, low self-esteem, or suicidal thoughts [3]. Depressive disorders can be masked by pain from other organs [4]. Patients define depression as “suffering of the soul and body” [5].

WHO estimated that approximately 350 million people worldwide suffer from depression [6], in Europe about 21–30 million people [7]. Depression is diagnosed twice as often in women (20–25%) than in men (7–12%) [8,9]. The reason for the difference in the incidence of depression between the sexes is not exactly known [10]. According to psychiatrists and sociologists, this may be due to men and women performing different social roles [11]. Women are at risk of developing depression during adolescence, before menstruation, during pregnancy, after delivery, and at perimenopausal age [12].

Depression in pregnancy is a significant public health problem; pregnancy and childbirth are some of the factors that contribute to the development of depression [13]. The incidence of depression in pregnant women varies depending on the current trimester [14]. Bennett et al. [15] based on a systematic review of studies on the development of depressive disorders in pregnant women estimated that in the first trimester depression occurs in 7.4% (2.2–12.6%) women, in the second trimester in 12.8% (10.7–14.8%), and in 12.0% (7.4–16.7%) of pregnant women in the third trimester. According to various studies, the incidence of depression in pregnancy ranges from 6–25% [16,17,18,19,20,21,22,23,24]. The diagnosing of depression is still an imperfect system. This is related to the similarity of the symptoms of depression to somatic disorders occurring during pregnancy [25]. For making a correct diagnosis the following symptoms, among others, are being used: a lack of interest in pregnancy, suicidal thoughts, and anhedonia [26]. Other factors of depression include, e.g., postpartum depression after previous deliveries, the occurrence of depression in the family, a pregnancy at a young age, an unplanned pregnancy, a previous miscarriage, a lack of or limited support of the environment and partner, conflicts with the father of the child, a low level of education, lack of work, and substance abuse [27].

In the first trimester of pregnancy, a rapid transformation of the hormonal system starts to occur (an increase in the number of estrogen and progesterone receptors) [28]. Estradiol and progesterone affect the neurotransmitter system of serotonin, dopamine and norepinephrine, causing emotional disorders [29]. In addition, women are afraid of having a miscarriage [30]. In the second trimester there is usually a stabilization of emotions. The last trimester of pregnancy is characterized by a renewed increase in the level of anxiety and uncertainty due to the approaching delivery. Due to changes in external appearance the physical self-esteem of women decreases, which also influences the development of depression [29].

It should be kept in mind that depression reduces the quality of life of pregnant women and significantly reduces the ability to provide proper care for their newborns [31]. Depression during pregnancy can also cause psychological problems in early childhood, poor academic performance, and impaired social functioning [32]. In addition, according to WHO data, depression during pregnancy is a strong risk factor for the development of postnatal depression [33], which may affect 10–15% of women in the period of up to 12 months after delivery [34]. Moreover, a lack of proper treatment of depression in an expectant mother may have a negative impact on the fetus (e.g., premature delivery, reduced birth weight, lower Apgar scores, as well as an increase in the concentration of stress hormones in the child) [35]. Early and correct diagnosis can minimize the negative effects of depression on both the mother’s and child’s health [36].

Considering the above, it seems to be particularly important to look for solutions that can safely minimize the risk of developing depression. One of such solutions may be a physical activity. Researches have shown that exercises performed during pregnancy have a positive impact on the health of mother and child [37], regular physical activity also minimizes the risk of developing depression [38]. WHO recommends 150 min of moderate physical activity or 75 min of intense training per week [39]. Unfortunately, despite many positive recommendations and guidelines regarding physical activity during pregnancy, currently less than 15% of pregnant women are physically active for a minimum of 150 min during the week [40].

The aim of this systematic review was to present the relationship between physical activity and the occurrence of depressive disorders during pregnancy. In particular, the goal was also to draw attention to both the type and intensity of physical activity that could potentially affect the prevention or minimization of depressive symptoms.

## 2. Materials and Methods

The review of the literature was carried out in the Medline-PubMed database. The basic search terms were: “pregnancy” AND “physical activity AND depression”.

### Search Strategy

“pregnancy”[All Fields] AND ((“exercise”[MeSH Terms] OR “exercise”[All Fields] OR (“physical”[All Fields] AND “activity”[All Fields]) OR “physical activity”[All Fields]) AND (“depressive disorder”[MeSH Terms] OR (“depressive”[All Fields] AND “disorder”[All Fields]) OR “depressive disorder”[All Fields] OR “depression”[All Fields] OR “depression”[MeSH Terms])) AND (“loattrfull text”[sb] AND (“2000/01/01”[PDAT]: “2018/12/31”[PDAT]) AND English[lang]). The work includes only English-language publications published in the years 2000–2018. The PRISMA (Preferred Reporting Items for Systematic Reviews and Meta-Analyses) principles were followed in the article overview.

For the analysis of titles, abstracts and full texts, set inclusion and exclusion criteria. The review included works that were published in the years 2000–2018 in English. The research should have been carried out on pregnant women or women that were pregnant or in puerperium; it should examine the relationship between physical activity and depression in pregnancy. Exclusion criteria include publications in a language other than English and studies not related to pregnant women. Studies that were carried out among pregnant women or women that were pregnant or in puerperium, but did not investigate the impact of physical exercise on depressive disorders during pregnancy were also rejected. In addition, the study did not include studies describing the relationship between physical activity and depression in pregnancy and puerperium or in the puerperium only in which it was impossible to clearly determine the effect for the period pregnancy. Systematic reviews, master’s theses, doctoral theses, letters to the editor were excluded. The study also excluded studies in which a high-risk pregnancy was diagnosed. Other exclusion criteria were: no access to the full version of the articles, no inclusion and exclusion criteria from the research available, inaccurate interpretation of the results and lack of a standardized scale as a research tool to assess depression. The correctness of the adopted inclusion and exclusion criteria has been verified by two independent researchers.

## 3. Results

**Figure 1:**
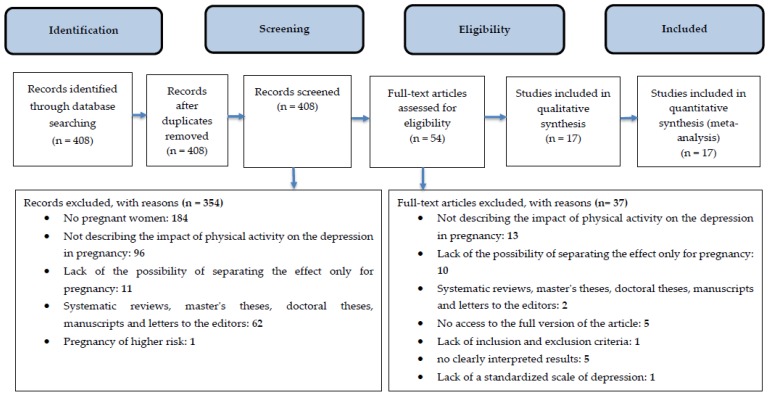
A diagram showing the stages of the literature review (2009 PRISMA flow diagram).

A total of 408 references were found; 354 articles were rejected on the basis of an analysis of titles, abstracts, and the language of publication. A total of 54 articles were left to read in full. Finally, these references were included in the review (Figure 1). The most important features of articles meeting the inclusion and exclusion criteria are described in Table 1.

After analysing the titles and abstracts of articles on the basis of criterion no. 2 (studies not related to pregnant women) 184 works were rejected. A total of 96 articles presented studies that were conducted among pregnant women or pregnant and in puerperium, but did not investigate the impact of physical exercise on depressive disorders during pregnancy (criterion no. 3). In addition, 11 papers were not included because they were studies describing the relationship between physical activity and depression in pregnancy and puerperium, or in the puerperium only, in which it was impossible to clearly determine the effects for pregnancy (criterion no. 4). The criterion of rejection no. 5 was met in 62 papers (systematic reviews, master’s theses, doctoral theses, letters to the editor), while in one article, the study group were high-risk pregnant women (criterion no. 6)

After the first stage of the review, 54 articles remained to be read in full. In 13 works, the study group were pregnant women, however, the impact of physical exercise on depression during pregnancy was not analysed (criterion no. 3). The lack of the possibility to clearly determine the effects of physical activity on the occurrence of depressive disorders during pregnancy was demonstrated in 10 articles (criterion no. 4). Two works were systematic reviews (criterion no. 5). In the case of five articles, their full version (criterion no. 7) was not accessible. In one study the inclusion and exclusion criteria (criterion no. 8) were not taken into account. Moreover, in five articles, the obtained results were interpreted in an unclear way, and in one study the standardized scale was not used to study depressive disorders (criteria nos. 9 and 10, respectively).

### 3.1. Supervised Physical Activity and Depression

Perales et al. [41] showed that regular, supervised physical activity significantly affects the development and severity of depression in pregnant women (n = 90). He tested the model of supervised training consisted of 55–60 min sessions, three times a week and lasted from 9–12 weeks of pregnancy to the end of the third trimester. Pregnant women from the control group did not perform any exercises. Post-cycle evaluation showed a significant reduction in the severity of depressive disorder in exercising, pregnant women compared to physically inactive women (7.67 ± 6.30 and 11.34 ± 9.74, respectively). In addition, in the group of women that were exercising the percentage of women who were diagnosed depressed on the basis of the CES-D scale (the Center for Epidemiologic Studies Depression Scale) significantly decreased (respectively from 22.4% before the project started to 12.2% after its completion). Among non-exercising women there was an increase in the frequency of diagnoses of depression (from 22.1% to 24.7%).

Similar studies were conducted in 2016 by El-Rafie et al. [42] on a group of 100 pregnant women. Depressive disorders were examined on the first day of the project and after 12 weeks of supervised training (three times a week for 60 min). The control group did not attend any physical activities. After 12 weeks of regular training, physically active women achieved significantly lower CES-D scores compared to non-exercising pregnant women (14.8 ± 5.3 and 20.0 ± 6.7, respectively). In addition, among the exercising women, there was a significantly lower intensity of depressive disorders during the study period (20.2 ± 6.4 to 14.8 ± 5.3, comparing the results before and after 12 weeks of training), which was not observed in physically inactive women (respectively 20.0 ± 6.7 before and 20.0 ± 6.7 after the end of the period under consideration). Studies on the effect of aerobic training on depressive disorders in nulliparous women [43] also showed a significant reduction in the degree of depressive disorders (CES-D scale) measured after a 3-month supervised physical training (3 times a week for 60 min). The training was conducted from 16–20 weeks to 28–32 weeks of pregnancy. After three months of training in physically active women, an improvement of four points in the CES-D scale compared to the inactive group was noted. Also Vargas-Terrones et al. [44] showed a significant decrease in the percentage of women with depressive disorders after participation in training sessions (18.6% vs. 35.6%). In these studies, training was conducted in the period from 12–16 weeks of gestation to the end of the third trimester, three times a week for 60 min each. Moreover, in the group of non-exercising pregnant women the percentage of women with depressive disorders increased from 18.5% to 35.6%. In the 38th week of the project, the percentage of pregnant women with depression from the physically active group was significantly lower (18.6%) compared to the inactive group of women (35.6%).

The study showed that physically active pregnant women are less likely to develop depression than physically passive women.

### 3.2. Marching Training

In 2016, Petrovic et al. [45] conducted a study on the influence of walking training on depressive disorders in women at 9 months of pregnancy (n = 200). It has been shown that with the increase of time devoted to physical activity, the level of depressive disorders decreases (*p* < 0.05; r = −0.14). In addition, the feeling of anxiety in pregnant women increases with increasing depression symptoms. The influence of long-term walking on the severity of depressive disorders in pregnant women was also studied [46]. A total of 118 women who were in the 30th week of pregnancy and had a sedentary lifestyle were included in the project. In part of women 30 min of brisk walking a minimum of three times a week was added to their everyday activities, where the others women were continue their current lifestyle. A questionnaire study was carried out at the beginning of the project (30 weeks of pregnancy) and at 32, 34, 36, and 38 or until delivery. The mood was rated using the POMS (the Profile of Mood States) scale. Based on the obtained results, it was observed that depressive disorders significantly decreased in walking group (*p* = 0.003). Similar results were not observed in the control group with no additional activity (*p* = 0.174).

The above studies show that unattended walking also has an impact on minimizing depressive disorders in pregnant women. It is worth noting that even 30 min of walking three times a week can significantly minimize depressive disorders in pregnant women.

### 3.3. Joga

During pregnancy activities that are soothing and calming to the mother’s organism are also very important. Yoga is an ideal relaxation workout for pregnant women. It combines elements of exercise, breathing, and meditation. It has been shown that, in relation to the general population, yoga significantly reduces stress [47]. In 2016 Kusaka et al. [48] examined the influence of supervised yoga training carried out between the 20th week of pregnancy and delivery on the mood of pregnant women (n = 60). The training was performed twice a month in a hospital. In addition, every pregnant woman was obliged to practice yoga three times a week at home. Effectiveness of yoga trainings was measure twice: between 27 and 32 weeks of pregnancy, and 34 and 37 weeks. The POMS scale results were significantly improved after both period, and the percent of women with depression decreased from 65.9% to 57.1% on the end of the project. Yoga has had a positive effect on such mood states as anxiety, anger, depression, fatigue, and embarrassment. In addition, after training, the energy level in daily physical activities increased significantly.

The effectiveness of yoga training on depressive disorders of pregnant women was also examined by Davis et al. [49]. In an 8-week program 23 pregnant women attended to a supervised, 75 min yoga training once a week. The control group was formed by 23 non-exercising women, who could use all methods of treatment outside the project. Questionnaires regarding depression, anxiety, negative emotions and physical activity were carried out on-line every week. After eight weeks of yoga training, the level of depression significantly decreased in group I (exercising women) from 10.13 to 6.37 and in group II (non-exercising women) from 10.57 to 7.32, but there was no statistically significant difference between the groups.

Yoga can be used as therapy for the treatment of depression in pregnant women. Regular training has an impact on reducing both depression and anxiety or other negative mood states. It is worth paying attention to the fact that yoga is a calming and relaxing activity. Mastering breathing skills and self-control will be very helpful during childbirth.

### 3.4. Physical Activity, Obesity, and Depression

The impact of physical activity on mental well-being was also examined among obese pregnant women [50]. The examined women (n = 98) were obliged to wear an accelerometer for at least three days (from getting up until going to sleep) in the remaining time, physical activity was described in a diary. Mental health was assessed using the WHO-5 scale (the WHO well-being index). Depression was found among 27% of the surveyed women, in whom the level of physical activity was significantly lower than in women without depression. Pregnant women with a positive frame of mind were active by 85% more minutes during the day compared to women with depressive disorders [50]. Claesson et al. [51] also examined the impact of physical activity on mental health of obese pregnant women. Using the EPDS scale (the Edinburgh Postnatal Depression Scale), he was assessing depressive disorders in the 11th and 35th week of pregnancy in groups of pregnant physically active and inactive women. He noticed a decrease in mean value from 5.5 ± 3.8 to 4.6 ± 3.7 in active pregnant women, while in non-active women this value changed from 7.1 ± 5.7 in the 11th week of pregnancy to 6.9 ± 5.1 in week 35. This study confirmed that moderate physical activity reduces the risk of depressive disorders in obese pregnant women.

The above studies show that regular physical exercise of moderate intensity has an impact on reducing the symptoms of depression.

### 3.5. Other Physical Activities

Regular physical activity is important both during pregnancy and before becoming pregnant. Tendais et al. [52] examined physical activity of women three months before pregnancy, at 10–15 and 19–24 weeks of pregnancy. Physical activity was assessed by the GPAQ questionnaire (the Global Physical Activity Questionnaire), depression by the Edinburgh scale. It was shown that during pregnancy the amount of time devoted to physical activity decreased significantly. Before pregnancy, recommendations for physical activity according to ACSM (American College of Sports Medicine) and AHA (American Heart Association) were met by only 14.3% of surveyed women. Physical activity recommendations according to ACOG (American College of Obstetrics and Gynecologists) were met in the first and second trimester by 12.5% of women each. The average level of depressive disorders was constant in both trimesters and there were no statistically significant differences between depressive disorders and the level of physical activity. During pregnancy, many women decide to limit physical activity and spend their free time passively. The relationship between the influence of physical activity and a sedentary lifestyle on depressive disorders was also examined [53]. The measurement was performed at 26–28 weeks of pregnancy using a questionnaire (n = 1144). Women with a higher level of physical activity were less prone to depressive disorders compared to women with low physical activity (OR 0.54, 95% CI 0.31–0.04, *p* = 0.03). A similar relationship was demonstrated in anxiety disorders. There were no statistically significant differences between a sedentary lifestyle and depression during pregnancy. According to U.S. Health and Human Services pregnant women should be physically active for a minimum of 150 min per week [54]. In a study of 820 pregnant women [55], it was shown that 25.9% of subjects had symptoms of mild depression and 19.1% of severe depression. Physical activity during early pregnancy was not associated with depression in mid-to-late pregnancy. Gjestland et al. [56] studied the impact of physical activity on the development of depressive disorders among 2753 pregnant women. The level of physical activity was tested at 17–21 weeks of pregnancy. A total of 1/3 of the surveyed women exercised less than 1× per week, 1–2× per week was practiced by 40% of pregnant women, and 26.6% were physically active at least 3× within a week. It was observed that physical activity at a level of 1–2× a week significantly reduces depressive mood disorders. A similar relationship was not recorded for an activity equal to or more than 3× a week. In 2011 [57] the relation between physical activity and depression was assessed among 1220 pregnant women by Demisse et al. It was reported that women with an MVPA ≤ 2.67 were less likely to develop depressive disorders compared to physically inactive pregnant women. Lower chances were also observed in women with a higher MVPA level. In an adjusted model, women with physical activity at the level of >9 MET-hours/week of total MVPA had a 31% lower risk of developing depression compared to pregnant women showing a lower level of activity. Omidvar et al. [58] examined the influence of five psychosocial factors (anxiety, stress, depression, dissatisfaction with marriage, social support) on aspects of lifestyle in pregnant women (nutrition, physical activity, caring for health, stress management, interpersonal relations and self-realization). Depression has been shown to adversely affect all six lifestyle factors in pregnant women. Takahasi et al. examined the influence of the lack of physical activity on the occurrence of depressive disorders, anxiety and stress levels. The study involved 1447 women currently in the 2nd trimester of pregnancy. A total of 39.8% of the surveyed women were physically inactive. Depressive disorders were found among 28.8% of pregnant women. There were no statistically significant differences between the lack of physical activity and the occurrence of depression among women in the 2nd trimester of pregnancy [59].

## 4. Discussion

Depression in pregnant women is a threat not only to the mother’s health, but also to the health of the child [60]. Depressive disorders may affect the child from the fetal stage, may also affect the course of labour, as well as the mother-child relationship in subsequent years [57]. During pregnancy, the ways of treating depression are limited. The use of antidepressants is not recommended, while physical activity [61] or psychotherapy [62] is can be a good way of therapy. Psychotherapy can also be used by women planning to become pregnant as an alternative to pharmacological treatment. In addition, both psychotherapy [63] and physical activity in pregnancy [64] effectively reduce the risk of depression in the perinatal period. An important issue is also the fact that among physically active pregnant women there is a higher rate of pregnancies with a normal course [65]. Physical activity also allows for the emotional balance to be regained [66]. Regular exercises help maintaining adequate mental and physical health. The level of depression and stress can be significantly reduced by 30-min training sessions held 5 times a week [67]. Unfortunately, many women still significantly reduce their level of physical activity after conception [68].

Despite the seriousness of the problem of depression in pregnancy, still very few papers assess the impact of physical activity in pregnancy on the development of depression in this period [41,42,43,44,45,46,48,49,50,51,52,53,55,56,57,58,59]. However, the results of this review clearly suggest that even a small amount of physical activity during pregnancy may reduce the severity of depressive symptoms, as well as the occurrence of depression. Research shows that the best forms of activity for a pregnant woman are walking, yoga, swimming [69] and general exercises (e.g., breathing, posture, and Kegel exercises) [70]. However, it should be remembered that the physical capacity of pregnant women varies in individual trimesters [71].

It seems important to note that even regular walks carried out during pregnancy, that are low-intensity exercises, can significantly reduce the symptoms of depression in pregnant women, as Petrovic et al. confirmed in their study [45]. This is in line with similar studies conducted by Taniguchi et al. [46]. In addition, it was observed that marching is an easy aerobic exercise for women who have so far lead a sedentary lifestyle, which significantly increases the availability of this form of activity. An effective alternative to walks may also be participation in supervised training sessions dedicated to this group of women. However, it should be borne in mind that for the safety of the pregnant woman and the child, all activities should be consulted with a gynecologist [72]. Research on the influence of supervised training on depressive disorders shows that aerobic exercises performed three times a week for about 60 min can significantly reduce the symptoms of depression in pregnant women [41,42,43,44]. Although there are also reports indicating that physical activity already at the level of 1–2 sessions a week may also be beneficial in reducing the frequency and severity of depressive symptoms in pregnant women Gjestland et al. [56]. When looking for safe and effective forms of physical activity, which may reduce the intensity and frequency of depressive disorders one should also pay attention to yoga trainings, as described in detail by Kusaka et al. [48] and Davis et al. [49]. These authors pointed out that yoga itself, as well as associated with any other form of physical activity, significantly improves the mental condition of pregnant women, reducing the symptoms of depressive disorders in the 2nd and 3rd trimester of pregnancy. It is also important to point out that women who are physically active in pregnancy have not only a lower risk of developing depression in pregnancy, but also in early and late puerperium [44]. In addition, Vargas-Terrones et al. [44] showed that women who do not exercise are more at risk of developing depressive disorders, both during pregnancy and postpartum compared to exercising women [44]. The role of physical activity as primary and secondary prevention of depression in pregnancy was also noted by El-Rafie et al. [42].

The positive effect of physical activity on depressive disorders can be explained by biological mechanisms. Exercises cause an increase in human body temperature. At the same time, the temperature of the brain increases, which results in a feeling of general relaxation and tranquility. In addition, after exercise, there is an increase in the level of b-endorphins, which are also responsible for a more positive frame of mind. Depression reduces the amount of neurotransmitters, such as serotonin, norepinephrine, or dopamine, which concentration increases after physical activity [41]. In addition, exercises prevent cardiovascular disorders, improve the functioning of the musculoskeletal system [73], increase appetite, improve the quality of sleep, and oxygenate the body better [69].

It is worth paying attention to the fact that during pregnancy there is a sudden and large increase in the size and weight of the body, while physical activity prevents overweight and/or obesity [74]. However, the observed changes in the external appearance of pregnant women, repeatedly reduce their self-esteem. Low self-esteem is a risk factor for both depression during pregnancy and postpartum [29]. The impact of physical activity on mental well-being was also examined among obese pregnant women [50,51]. Claesson et al. [51] noted that physical activity in obese women not only reduces the risk of developing depression, but also increases their quality of life. These results become particularly important in the light of reports by De Wit et al. [50], who showed that obese pregnant women often have a depressed mood, which in consequence affects the restriction of physical activity. Although Tendais et al. [52] have observed that physical activity improves the overall quality of life of pregnant women, it does not significantly reduce depressive symptoms. Omidvar et al. [58] have shown its effect not only in minimizing the symptoms of depression but also on anxiety. A similar result was also obtained in the studies of Padmapriya et al. [53]. Demisse et al. [57] have shown that women who are physically active in pregnancy are less likely to develop depression compared to inactive women.

With the above in mind, it can undoubtedly be said that mental and physical health are dependent on each other. It is worth to educate pregnant women about a healthy lifestyle which also includes physical activity. The time of pregnancy, childbirth and then raising a child is one of the most beautiful periods of life for every woman. It is a period when the well-being of women affects not only themselves, but also directly their child, while physical activity significantly raises the energy level of pregnant women, which is associated with a lower mood depression. Physical activity as a part of a healthy lifestyle [37], plays an important role in maintaining the proper psychophysical state of a pregnant woman [40]. In this paper we summarized the positive influence of regular, moderate physical activity on depressive disorders in pregnant women, demonstrating their important role in improving the mood/well-being of pregnant women as well as reducing the incidence of depression. It can be noted that in the studies in which the type of physical activity was carefully recommended and performed both under supervision and without supervision results of a statistically significant impact of exercises on reducing symptoms of depression were achieved. In all studies, the authors pointed out that physical activity is an important element of a healthy lifestyle during pregnancy. However, remember that depression is affected not only by the behaviour of pregnant women, but also by their surroundings.

## 5. Conclusions

Based on this review, it can be concluded that physical activity reduces the symptoms of depression during pregnancy and can be a form of safe preventive treatment. Physically active women (before and/or during pregnancy) had a lower risk of developing depression compared to non-active women. In addition, exercise has had an impact on reducing both the level of anxiety and stress, as well as the overall quality of life. It should also be noted that introducing physical activity during pregnancy or before will not always protect the pregnant woman against the development of depression or affect its course during pregnancy. This suggests the existence of a multifactorial dependency, where physical activity is only one its components.

## Figures and Tables

**Table 1:** Study characteristics.

First Author (Year), Country	Title of Article	Main Objective	Design	Sample Demographics	Research Tool	Main Results
Davis K. et al. (2015), USA	A randomized controlled trial of yoga for pregnant women with symptoms of depression and anxiety	The effects of yoga on depressive and anxiety disorders in pregnant women	Randomized controlled trial	N = 46: Yoga (n) = 23 TAU (n) = 23	Questionnaire of *Demographics and mental health history*, *Structured clinical interview for DSM disorders research version SCID-RV*, *Yoga diary*, *Treatment tracking sheet*, *International physical activity questionnaire (IPAQ)*, *Client satisfaction questionnaire (CSQ-8)*, *Credibility scale*, *The Edinburgh perinatal depression scale (EPDS)*, *The state-trait anxiety inventory (STAI)*, *The positive and negative affect schedule-negative subscale (PANAS-N).*	The level of depressive disorders decreased in the group of women practising yoga as well as in women in the control group. Pregnant women from the control group could use all forms of treatment for depression outside the project. A greater decrease in depressive symptoms was observed in women from the group in which yoga was used as an intervention
Robledo-Colonia A. F. et al. (2012), Colombia	Aerobic exercise training during pregnancy reduces depressive symptoms in nulliparous women: a randomised trial	The effects of a 3-month supervised breathing training on depressive disorders in the nulliparous women	Randomized controlled trial	N = 74 Exp. gr. = 37 Con. gr. = 37	*The Center for Epidemiological Studies-Depression* *Scale (CES-D)*	Breathing exercises significantly reduced depressive symptoms in pregnant women compared to non-exercising women
Petrovic D. et al. (2016), Serbia	Association between walking, dysphoric mood and anxiety in late pregnancy: A cross-sectional study	The relationship between anxiety, depression and physical activity in women at 9 months of pregnant	Cross-sectional study	N = 200	*Questionnaire of Physical Activity*, *Beck Depression Inventory*, *Zung Anxiety Scale*	The level of depression and anxiety decreases with the increase in the level of physical activity. The level of anxiety disorders increases with the development of depression.
Padmapriya N. et al. (2016), Singapore	Association of physical activity and sedentary behaviour with depression and anxiety symptoms during pregnancy in a multi-ethnic cohort of Asian women	The influence of physical activity and a sedentary lifestyle on depressive and anxiety disorders in pregnant Chinese, Malaysian and Indian women	Cohort study	N = 1144	*The Edinburgh Postnatal Depression Scale (EPDS)*, *The Spielberger State-Trait Anxiety Inventory (STAI)*, *Questionnaire of Physical Activity and Sedentary Behavior*	A higher level of physical activity reduces the risk of depression and anxiety. There is no relation between time spent in a sitting position (e.g., watching TV) and depressive and anxiety disorders
El-Rafie M.M. et al. (2016), Egypt	Effect of aerobic exercise during pregnancy on antenatal depression	The effects of exercises on prevention and treatment of antenatal depression in pregnant women	Randomized controlled trial	N = 100 Exp.gr. = 50 Con.gr. = 50	*The Center for Epidemiological Studies-Depression* *Scale (CES-D)*	After 3 months of training, a significant decrease in depressive disorders was observed in exercising women compared to physically inactive pregnant women
Perales M. et al. (2015), Spain	Exercise During Pregnancy Attenuates Prenatal Depression: A Randomized Controlled Trial	The impact of supervised training on reducing depression in pregnant women	Randomized controlled trial	N = 167 Exp.gr. = 90 Con.gr. = 77	*The Center for Epidemiological Studies-Depression* *Scale (CES-D)*	Supervised moderate-intensity physical activity reduces the symptoms of depression. Exercises did not show any negative impact on the course of pregnancy.
Taniguchi S. et al. (2016), Japan	Home-based walking during pregnancy affects mood and birth outcomes among sedentary women: A randomized controlled trial	The impact of unsupervised walking training on depressive disorders in women who were not regularly physically active before becoming pregnant	Randomized controlled trial	N = 118 Exp.gr. = 60 Con.gr. = 58	*The Profile of Mood States (POMS)*, *Pedometer (WALKi’NZOKU* *WZ100*	Unsupervised walking training reduces depressive symptoms in pregnant women
Kusaka M. et al. (2016), Japan	Immediate stress reduction effects of yoga during pregnancy: One group pre–post test	The influence of yoga on stress and negative mood during pregnancy	Clinical trial study	N = 60	*The Profile of Mood States (POMS)*	The level of stress and negative emotions (including depression) decreased after yoga training at both measuring point I (27–32 weeks of pregnancy) and measuring point II (34–37 weeks of pregnancy)
Omidvar S. et al. (2018), Iran	Associations of psychosocial factors with pregnancy healthy life styles	The influence of the five psychosocial factors (depression, stress, anxiety, marital dissatisfaction, social support) on a healthy lifestyle (nutrition, physical activity, maintaining health, interpersonal relations, self-fulfilment) in pregnant women	Cross-sectional study	N = 445	*Health-Promoting Lifestyle Profile (HPLP II)*, *Beck Depression Inventory (BDI-II), Prenatal Distress Questionnaire (PDQ)*, *State-Trait Anxiety Inventory (STAI), Marital Satisfaction Scale (MSS)*, *Social Support Questionnaire (SSQ)*	Depression negatively affects all six aspects of a healthy lifestyle (nutrition, physical activity, maintaining health, interpersonal relations, self-fulfillment)
Demisse Z. et al. (2011), USA	Physical activity and depressive symptoms among pregnant women: the PIN3 Study	Association between physical activity and depressive symptoms during pregnancy	Cohort study	N = 1220	*Questionnaire about physical activity, accelerometer*, *a physical* *activity diary* *The Borg Scale*, *The Center for Epidemiologic Studies Depression* *Scale (CES-D)*, *The Life Experiences Survey (LES)*, *The Medical Outcomes Study Social Support Scale*	Women with physical activity at levels above zero to 2.67 total MVPA had a lower risk of depression compared to non-active women. A similar relationship was reported in women with higher MVPA. Women who had physical activity at >9 MET hours/week of total MVPA were 31% less likely to have depressive disorder compared to less active women
De Wit L. et al. (2015), The Netherlands	Physical activity, depressed mood and pregnancy worries in European obese pregnant women: results from the DALI study	An evaluation of the relationship between the mental health status and physical activity	Cross-sectional study	N = 98	*Physical activity: Actigraph GT3X, GT1M or Actitrainer accelerometer*, *The WHO well-being index (WHO-5)*, *The Cambridge Worry Scale (CWS)*, *The Likert scale*	A lower level of physical activity is associated with a lowering of mood.
Claesson I.M. et al. (2012), Sweden	Physical activity and psychological well-being in obese pregnant and postpartum women attending a weight-gain restriction programme	Differences in mental well-being and the quality of life of obese pregnant physically active and inactive women.	Prospective intervention study	N = 153	*Physical exercises diary*, *The Edinburgh Postnatal Depression Scale (EPDS)*, *The 36-Item Short-* *Form Health Survey (SF-36)*, *Beck Anxiety Inventory (BAI)*	Moderate physical activity a minimum of 3x a week reduces the risk of depressive disorders in pregnant women
Gjestland K. et al. (2012), Norway	Do pregnant women follow exercise guidelines? Prevalence data among 3482 women and prediction of low-back pain, pelvic girdle pain and depression	The impact of physical activity on pain in the lumbar region of the spine, pelvis and depressive disorders at 32 weeks of pregnancy	Cohort study	N = 2753	*4 questionnaires about physical activity, low-back pain, pelvic girdle pain*, *The Edinburgh Postnatal Depression Scale (EPDS)*	Physical activity 1–2× a week significantly reduces depressive symptoms in pregnant women. A similar relationship was not recorded for physical activity over 3× a week
Vargas-Terrones M. et al. (2018), Spain	Physical exercise programme during pregnancy decreases perinatal depression risk: a randomized controlled trial	An evaluation of the impact of exercise on the occurrence of depression during pregnancy	Randomized controlled trial	N = 124 Exp.gr. = 70 Con.gr. = 54	*The Center for Epidemiologic Studies Depression* *Scale (CES-D)*, *the Polar FT7 heart rate monitor*	Physical activity significantly reduces the level of depressive disorders in women exercising during pregnancy
Szegda K. et al. (2018), USA	Physical activity and depressive symptoms during pregnancy among Latina women: a prospective cohort study	An evaluation of the impact of physical activity on the development of depression in the Latino population of pregnant women, at high risk of developing depressive disorders	Prospective cohort study	N = 820	*The Pregnancy Physical Activity* *Questionnaire (PPAQ)*, *The Edinburgh Postnatal Depression Scale (EPDS)*, *Cohen’s* *Perceived Stress Scale*, *The State-Trait Anxiety Survey*	Physical activity does not affect the increase of depressive disorders in pregnant Latina women
Tendais I. et al. (2011), Portugal	Physical activity, health-related quality of life and depression during pregnancy	An evaluation of the relationship between physical activity and the quality of life conditioned by the state of health and depressive disorders during pregnancy	A longitudinal study	N = 56	*The 36-Item Short-* *Form Health Survey (SF-36)*, *The Global Physical Activity Questionnaire (GPAQ)*, *The Edinburgh* *Postnatal Depression Scale (EPDS)*	The patterns of physical activity depend on the stage of pregnancy. Both physical and mental health components are different in pregnancy, regardless of physical activity
Takahasi E. H. M. et al. (2013), Brasil	Mental health and physical inactivity during pregnancy: a cross-sectional study nested in the BRISA cohort study	The relationship between mental health and a lack of physical activity in women in the 2nd trimester of pregnancy	A cross-sectional study	N = 1447	*The International Physical Activity* *Questionnaire (IPAQ)*, *The Center for Epidemiologic Studies Depression* *Scale (CES-D)*, *The Beck Anxiety Scale (BAI)*, *The Perceived Stress Scale (PSS-14)*	There is no statistically significant relationship between symptoms of depression and stress and the lack of physical activity.
